# Natural Antioxidant Enrichment of Olive Pâté: Effects of Microencapsulated Bergamot By-Product Extract Levels on Quality and Storage Stability

**DOI:** 10.3390/foods15172996

**Published:** 2026-08-26

**Authors:** Iolanda Cilea, Antonio Gattuso, Simone Santacaterina, Giorgio Vilardi, Amalia Piscopo, Alessandra De Bruno, Marco Poiana

**Affiliations:** 1 Department of AGRARIA, University Mediterranea of Reggio Calabria, 89124 Reggio Calabria, Italy; iolanda.cilea@unirc.it (I.C.); simone.santacaterina@unirc.it (S.S.); giorgiovilardi98@gmail.com (G.V.); amalia.piscopo@unirc.it (A.P.); mpoiana@unirc.it (M.P.); 2 Department of Human Sciences and Promotion of the Quality of Life, San Raffaele University, 00166 Rome, Italy

**Keywords:** bergamot by-products, *Citrus bergamia*, microencapsulated, natural antioxidants, Nocellara Messinese, olive pâté, oxidative stability, sensory quality

## Abstract

Olive pâté is a lipid-rich semi-solid product susceptible to oxidative deterioration during storage. This study investigated microencapsulated bergamot (*Citrus bergamia*) by-product extract (MBE) as a natural antioxidant for improving olive pâté stability. MBE, obtained by spray drying with 20% (*w*/*w*) maltodextrin, was incorporated at 2.5 or 5.0 g per 100 g of olives (OP_2.5_ and OP_5_), while a non-enriched formulation served as control (CTR). Samples were stored at 20 and 30 °C for 100 days and analysed for physicochemical, colour, phenolic, antioxidant, microbiological, sensory, and oxidative stability. Storage temperature and formulation significantly influenced quality evolution. At 30 °C, OP_2.5_ showed the lowest total colour difference after 100 days (ΔE* = 3.29 versus 5.05 for CTR) and the highest final DPPH radical-scavenging activity. Both enriched formulations exhibited longer Oxitest induction periods than CTR at day 0 (26.53 and 25.33 vs. 19.85 h) and after 100 days at 30 °C (22.30 and 21.50 vs. 16.45 h). Microbial counts remained low, while sensory profiling indicated limited rancidity development and preservation of the main sensory characteristics. MBE effects were not proportional to the amount added, with OP2.5 providing the most balanced performance. These findings support MBE as a promising clean-label ingredient for improving olive pâté stability.

## 1. Introduction

Table olives (*Olea europaea* L.) and their derived products are widely appreciated as key components of the Mediterranean diet owing to their favourable lipid profile, low carbohydrate content, and high concentration of bioactive compounds, particularly phenolic antioxidants, which contribute to both nutritional value and oxidative stability [1,2]. Southern Italy is one of the main olive-growing regions of the Mediterranean area and is characterized by a rich diversity of cultivars with distinct technological and compositional traits [3,4]. Among these, the cultivar Nocellara Messinese is particularly valued for its sensory characteristics and phenolic composition. Previous studies on monovarietal table olives and virgin olive oils have demonstrated that cultivar significantly influences phenolic composition, antioxidant capacity, and oxidative stability [5], suggesting that these cultivar-dependent characteristics may also affect the technological performance and shelf-life of processed products such as olive pâté.

Olive pâté is a semi-solid spread prepared from processed table olives, generally blended with vegetable oil and seasonings and subsequently pasteurized to ensure microbiological safety and extend shelf-life [6,7]. Compared with intact olives, the homogenization process disrupts plant tissues, increasing oxygen exposure and accelerating oxidative reactions within the lipid-rich matrix. Consequently, despite thermal stabilization, olive pâté remains susceptible to oxidative deterioration during storage, resulting in pigment degradation, loss of antioxidant compounds, development of off-flavours, and reduced consumer acceptance [8,9]. For this reason, the development of clean-label preservation strategies capable of maintaining both the nutritional and sensory quality of olive-based spreads has become increasingly important in response to the growing demand for minimally processed functional foods [10].

The replacement of synthetic preservatives with natural bioactive compounds recovered from agro-industrial by-products has attracted considerable scientific and industrial interest in recent years. Beyond improving food quality, this approach contributes to the valorisation of food-processing residues within a circular economy framework. Among citrus by-products, bergamot (*Citrus bergamia* Risso & Poiteau) pomace represents a particularly valuable source of flavanone glycosides, including neoeriocitrin, narirutin, naringin and neohesperidin, together with the characteristic 3-hydroxy-3-methylglutaryl-flavanones brutieridin and melitidin, compounds recognized for their antioxidant properties [11]. Nevertheless, the direct incorporation of plant extracts into food matrices may be limited by limited chemical stability, interactions with food components, reduced bioavailability, and undesirable sensory modifications, which can ultimately compromise their technological effectiveness [12,13,14].

Microencapsulation has emerged as an effective strategy to overcome many of these limitations by protecting sensitive compounds from environmental degradation, improving their stability during processing and storage, and modulating their release within the food matrix [12,13,14]. Several studies have demonstrated the potential of natural antioxidant ingredients, including encapsulated bioactive and agro-food by-product extracts, to improve the quality and stability of food products [12,15]. Indeed, antioxidant performance depends on multiple interacting factors, including release kinetics from the encapsulating matrix, interactions with endogenous phenolic compounds, partitioning between aqueous and lipid phases, and storage conditions. Consequently, increasing the concentration of encapsulated antioxidants does not necessarily result in proportional improvements in product stability [16].

Storage temperature is one of the major factors affecting the evolution of quality attributes in olive-based products during shelf life, influencing oxidative reactions, pigment stability and the preservation of bioactive compounds [9,17]. Evaluating the behaviour of food products under different, yet technologically realistic, storage temperatures provides useful information on the stability of food formulations and on the effectiveness of natural stabilizing ingredients under conditions representative of commercial distribution and storage [17]. However, despite the increasing interest in clean-label olive-based spreads, limited information is available on how storage temperature interacts with natural antioxidant enrichment in determining the physicochemical, antioxidant, microbiological and sensory evolution of olive pâté.

Despite the growing interest in the use of natural antioxidants in food applications, the effect of microencapsulated bergamot by-product extract concentration on the storage stability of olive pâté remains insufficiently investigated. The enrichment level is a critical factor, as it may influence antioxidant effectiveness, physicochemical properties, and sensory quality [18]. However, higher addition levels do not necessarily result in proportional improvements in overall product quality. Therefore, this study compared two enrichment levels and a non-enriched control in olive pâté produced from the Nocellara Messinese cultivar and stored at 20 and 30 °C. Colour, physicochemical, phenolic, antioxidant, microbiological, sensory, and oxidative stability parameters were monitored to identify the formulation providing the best balance between protection against deterioration and quality retention.

## 2. Materials and Methods

### 2.1. Raw Materials

Bergamot (*Citrus bergamia* Risso et Poiteau) pomace was supplied by Citrus Juices S.r.l. (Reggio Calabria, Italy). The pomace was dried at 50–60 °C using a tangential air-flow cabinet (“Scirocco” model, Società Italiana Essiccatoi, Milan, Italy) to a final moisture content of approximately 12% and subsequently milled (Vevor, Shanghai, China) into a homogeneous powder.

Naturally fermented table olives (*Olea europaea* L., cv. Nocellara Messinese) were used for pâté preparation. Before processing, the olives were naturally fermented in brine containing 8% (*w*/*v*) NaCl.

### 2.2. Preparation of Microencapsulated Bergamot Extract (MBE)

Bergamot pomace was extracted using an ethanol/water mixture (50:50, *v*/*v*) under magnetic stirring at 70 °C for 30 min, according to Gattuso et al. [11]. After extraction, ethanol was removed from the hydroalcoholic extract using a rotary evaporator (Heidolph WB 2000, Heidolph Instruments GmbH & Co. KG, Schwabach, Germany). The concentrated extract was subsequently reconstituted with distilled water to a concentration of 6 °Brix. This concentration was selected to limit caramelization phenomena and reduce operational problems during spray drying, particularly nozzle clogging and fouling.

The extract was microencapsulated by spray drying using two separate feed streams: the bergamot extract, used as the core material, and an aqueous maltodextrin solution (20%, *w*/*v*), used as the wall material. Spray drying was performed using a Büchi S-300 mini spray dryer (Büchi Labortechnik AG, Flawil, Switzerland) equipped with a triple-fluid nozzle. The operating conditions were as follows: inlet drying-air temperature, 130 °C; outlet temperature, 80 °C; atomizing-air volumetric flow rate, 1400 L; bergamot extract feed rate, 2 mL min^−1^; maltodextrin solution feed rate, 3 mL min^−1^; drying-air volumetric flow rate, 35 m^3^ h^−1^; and nitrogen as the atomizing gas. The resulting microencapsulated bergamot extract powder (MBE) was collected and stored in sealed amber glass containers at room temperature until further analysis.

### 2.3. Characterization of Microencapsulated Bergamot Extract (MBE)

#### 2.3.1. Moisture Content (MC) and Water Activity (a_w_)

Moisture content (MC) was determined using a MA37 moisture analyser (Sartorius, Göttingen, Germany) by drying approximately 1 g of sample at 105 °C. Results were expressed as percentage (%). Water activity (a_w_) was measured at 25 °C using an Aqualab LITE hygrometer (Decagon Devices Inc., Pullman, WA, USA). All measurements were performed in triplicate.

#### 2.3.2. Preparation of MBE for Phenolic and Antioxidant Analyses

For the extraction of total phenolic compounds from the MBE, 1 g of microcapsules was dissolved in 8.7 mL of acidified distilled water to pH 2.0 using 2 N HCl. The suspension was vortexed, sonicated for 50 min using a Sonoplus Ultrasonic homogeniser HD 2200.2 (BANDELIN electronic GmbH & Co. KG, Berlin, Germany), and centrifuged at 8000 rpm for 5 min at 4 °C. The supernatant was filtered and used for subsequent analyses.

#### 2.3.3. Total Phenolic Content (TPC) and Antioxidant Activity of MBE

TPC of MBE was determined according to Azarpazhooh et al. [19]. 0.05 mL of MBE was mixed with 6.0 mL of distilled water and 0.5 mL of Folin–Ciocalteu reagent. After 8 min of incubation at room temperature, 1.5 mL of sodium carbonate solution (20%, *w*/*v*) was added. The reaction mixture was incubated in the dark at room temperature for 30 min, and absorbance was measured at 765 nm using a dual-beam UV–Vis spectrophotometer (Perkin-Elmer UV–Vis λ2, Waltham, MA, USA). Quantification was performed using a gallic acid calibration curve (y = 0.0935x + 0.0024, r = 0.9986). Results were expressed as mg gallic acid equivalents per g of microencapsulated powder (mg GAE g^−1^).

Antioxidant activity was evaluated using DPPH and ABTS radical scavenging assays according to Sarabandi et al. [20].

For the DPPH assay, 2.60 mL of methanolic DPPH solution (6 × 10^−5^ M) was placed in a cuvette, and the initial absorbance (t_0_) was recorded at 515 nm. Subsequently, 40 µL of MBE was added. The mixture was kept under constant stirring in the dark at room temperature for 30 min, centrifuged at 6000 rpm for 5 min, and the absorbance of the supernatant was measured.

For ABTS assay, 2.80 mL of ethanolic ABTS solution (7 mM) was placed in a cuvette, and 20 µL of MBE was added. After 6 min of incubation in the dark at room temperature, the mixture was centrifuged (6000 rpm, 5 min) and absorbance was measured at 734 nm.

Antioxidant activity was quantified using a Trolox calibration curve (DPPH: y = 8.0686x − 2.2104, r = 0.9967; ABTS: y = 4.2147x − 2.4745, r = 0.9994) and expressed as mmol Trolox equivalents per gram of MBE (mmol TE g^−1^). All analyses were carried out in triplicate.

#### 2.3.4. UHPLC Analysis of MBE

Individual phenolic compounds (IPCs) in MBE were identified and quantified by UHPLC with photodiode array detection (UHPLC–PDA) according to Romeo et al. [21], with minor modifications.

Analyses were performed using a PLATINblue UHPLC system (Knauer, Berlin, Germany) equipped with a binary pump, autosampler, column oven, and PDA-1 detector, controlled by Clarity 6.2 software. Separation was carried out on a C18 reversed-phase column (Knauer, 1.8 µm, 100 × 2.0 mm). The column temperature was maintained at 30 °C. The injection volume was 5 µL. The mobile phases were water acidified to pH 3.10 with acetic acid (A) and acetonitrile (B). The gradient program was as follows: 0–3 min, 95% A and 5% B; 3–15 min, 95–60% A and 5–40% B; 15–15.5 min, 60–0% A and 40–100% B. The flow rate was 0.4 mL min^−1^.

Identification and quantification were performed by comparing retention times and UV spectra with those of external standards used to construct calibration curves. Results were expressed as mg g^−1^ DM.

#### 2.3.5. Scanning Electron Microscopy (SEM)

Morphological and microstructural features of microcapsules were examined using a Thermo Scientific Phenom XL G2 Desktop SEM (Ferentino, Italy), according to Zhu et al. [22]. Samples were mounted on double-sided carbon tape, gold-coated, and observed in SED mode at an accelerating voltage of 10 kV.

### 2.4. Olive Pâté Preparation

Olive pâté was prepared using naturally fermented table olives (*Olea europaea* L., cv. Nocellara Messinese). For each formulation batch, one kilogram of olives was homogenized with 100 mL of sunflower oil using a laboratory blender until a uniform paste was obtained. MBE was incorporated during homogenization at two enrichment levels, corresponding to 2.5 and 5.0 g per 100 g of olives. The resulting formulations were designated OP_2.5_ and OP_5_, respectively, whereas a formulation without MBE was used as the control (CTR).

The theoretical phenolic contribution of MBE was calculated from the dry matter content and total phenolic content of the microencapsulated powder. Based on these values, the theoretical phenolic contribution of MBE was 16.94 and 33.88 mg GAE per 100 g of olives for OP2.5 and OP5, respectively (Table 1).

**Figure 1:**
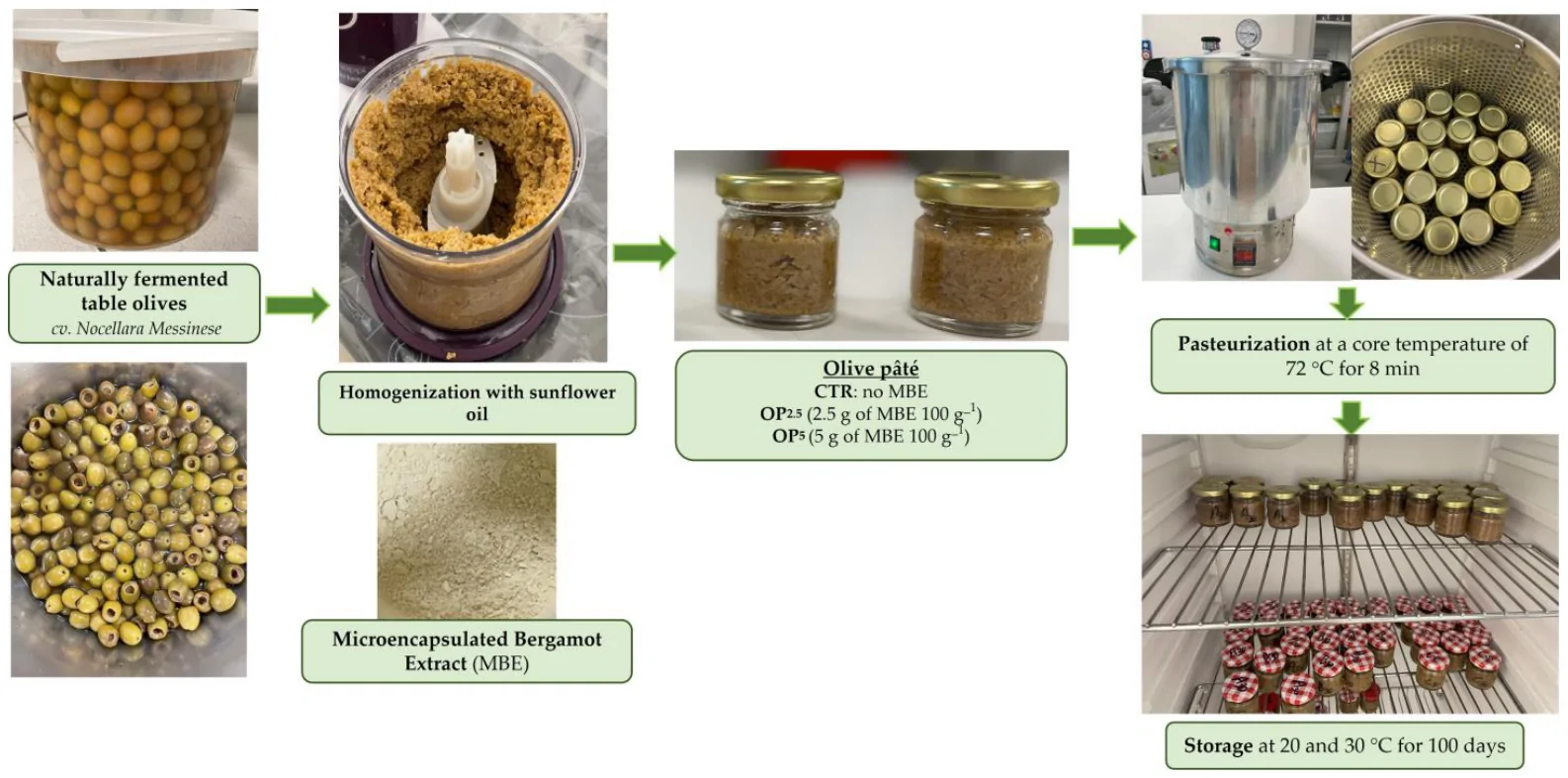
Experimental workflow for the preparation, pasteurization, and storage of olive pâté formulations enriched with MBE.

The pâté was filled into 40 g glass jars, hermetically sealed and pasteurized at 72 °C for 8 min, corresponding to the product core temperature monitored throughout processing. After pasteurization, jars were rapidly cooled to room temperature before storage. The overall preparation procedure is summarized in Figure 1.

### 2.5. Experimental Design and Storage Conditions

The experimental design included three formulations (CTR, OP_2.5_ and OP_5_), two storage temperatures (20 and 30 °C), and five sampling times (0, 15, 30, 60 and 100 days). Fresh pâté samples analysed immediately after pasteurization and cooling (t0) represented the common baseline for all formulations before storage. The remaining jars were then stored at either 20 or 30 °C in thermostatically controlled chambers. Sampling was destructive, and three independent jars were analysed for each formulation, storage temperature and sampling time (*n* = 3).

### 2.6. Quality Evaluation of Olive Pâté During Storage

Physicochemical, colour, antioxidant, sensory, and microbiological analyses were carried out on olive pâté samples during storage to evaluate the effects of formulation, storage temperature, and storage time on the main quality attributes of the product.

#### 2.6.1. Colour Analysis

Colour parameters (L*, a*, b*) were evaluated using a Minolta CR-700 automatic tristimulus colourimeter (Konica Minolta, Osaka, Japan), using the CIE Lab colour space [23]. Each pâté sample was evenly distributed in a glass vessel, and ten colour measurements were recorded at different points on the surface to account for sample heterogeneity.

The total colour difference (ΔE*) was calculated at each sampling time relative to the corresponding Day 0 value for each formulation and storage temperature, according to the following equation:

ΔE* = [(ΔL*)^2^ + (Δa*)^2^ + (Δb*)^2^]^1/2^

where ΔL*, Δa*, and Δb* represent the differences between each storage time and the corresponding initial value. ΔE* was calculated throughout storage and used as an integrated descriptor of overall colour change over time.

#### 2.6.2. pH Measurement

pH was determined according to Sánchez et al. [24]. Five grams of homogenized pâté were diluted in 25 mL of deionized water and mixed until a uniform suspension was obtained. The pH was measured using a calibrated pH meter (Crison Basic 20, Crison Instruments, S.A., Barcelona, Spain). Measurements were performed in triplicate at 25 °C at each sampling time for all formulations and storage conditions.

#### 2.6.3. Preparation of Pâté Extracts

Phenolic compounds were extracted from olive pâté samples according to Romeo et al. [21], with minor modifications. Briefly, 2 g of pâté were mixed with 2 mL of hexane and 10 mL of methanol/water solution (70:30, *v*/*v*). The mixture was homogenized using an Ultra-Turrax homogenizer (IKA T 25 digital ULTRA-TURRAX, Staufen, Germany). The hydroalcoholic phase was separated by centrifugation at 9000 rpm for 5 min at 4 °C using an NF 1200R centrifuge (Nüve, Ankara, Turkey). The recovered extract was filtered through a regenerated cellulose membrane before subsequent spectrophotometric and chromatographic analyses.

#### 2.6.4. Total Phenolic Content of Pâté

Total phenolic content (TPC) was determined according to De Bruno et al. [25], with minor modifications. Briefly, 300 µL of appropriately diluted pâté extract was placed in a 25 mL volumetric flask. Then, 2.5 mL of methanol/water solution (80:20, *v*/*v*), 0.625 mL of Folin–Ciocalteu reagent, and 1.250 mL of sodium carbonate solution (20%, *w*/*v*) were added. The samples were kept in the dark overnight at room temperature. Absorbance was measured at 725 nm using a UV–Vis spectrophotometer. Quantification was performed using a gallic acid calibration curve (y = 0.1204x + 0.002, r = 0.9993). Results were expressed as mg gallic acid equivalents per kg of pâté on a dry matter basis (mg GAE kg^−1^ DM).

#### 2.6.5. Antioxidant Activity of Pâté

Antioxidant activity of pâté extracts was evaluated using the DPPH radical scavenging assay according to De Bruno et al. [25], with minor modifications. Briefly, 2.980 mL of DPPH methanolic solution (6 × 10^−5^ M) was mixed with 20 µL of appropriately diluted pâté extract. The reaction mixture was incubated in the dark at room temperature for 30 min, and absorbance was measured at 515 nm. Results were calculated using a Trolox calibration curve (1.5–24 µmol) (y = 8.0686x − 2.2104, r = 0.9967) and expressed as µmol Trolox equivalents per g dry matter (µmol TE g^−1^ DM).

#### 2.6.6. UHPLC Analyses of Pâté Phenolic Compounds

Identification and quantification of individual phenolic compounds in pâté extracts were performed by UHPLC–PDA, following the chromatographic conditions described for the microencapsulated bergamot extract in Section 2.3.4. Results were expressed as mg g^−1^ DM.

#### 2.6.7. Oxidative Stability Study

Oxidative stability was evaluated using the Oxitest method according to De Bruno et al. [26]. Ten grams of olive pâté were placed in a sealed titanium chamber pressurized with oxygen to 6 bar and heated at 90 °C. The induction period (IP), defined as the time required to reach the onset of lipid oxidation, was determined from the decrease in oxygen pressure caused by oxygen consumption during the oxidation process. The IP was automatically calculated using OXISoft™ software (version 10002948, Usmate Velate, MB, Italy) and expressed in hours.

#### 2.6.8. Microbiological Analyses

Microbiological analyses were performed according to Panagou [27], with modifications. Five grams of pâté sample were homogenized in Ringer solution at a 1:10 ratio using a stomacher (BagMixer^®^ 400 P, Interscience, Saint-Nom-la-Bretèche, France). Serial dilutions were prepared and plated on Plate Count Agar (PCA) for total aerobic bacteria (CBT), incubated for 48 h at 25 ± 2 °C, and on Dichloran Rose Bengal Chloramphenicol (DRBC) agar for yeasts and moulds, incubated for 4–5 days at 25 ± 2 °C. Results were expressed as log10 CFU g^−1^.

#### 2.6.9. Sensory Analysis

Sensory evaluation was performed on freshly prepared olive pâté samples (t0) and after 100 days of storage at 20 and 30 °C. The assessment was carried out by a trained panel consisting of ten assessors aged between 20 and 45 years and experienced in the sensory evaluation of olive-based products. According to institutional procedures, formal approval by a bioethics committee was not required for this type of sensory evaluation. All assessors received information regarding the study procedures, ingredients, and potential risks, and provided written informed consent prior to participation. Only samples considered microbiologically suitable for consumption were subjected to sensory evaluation.

At each evaluation session, all assessors evaluated the three formulations (CTR, OP_2.5_, and OP_5_). Samples were served at room temperature and presented to each assessor in a randomized order. Each sensory assessment was performed in duplicate.

Fifteen sensory attributes covering appearance, aroma, taste, texture, and overall quality were assessed. Appearance attributes included green colour, homogeneity of colour, and brown colouration. Aroma-related attributes included fermented smell and aromatic intensity. Taste-related attributes comprised salty, acidic, bitter, sweet, vegetable note, rancidity, and aftertaste. Textural attributes included greasiness and creaminess. Overall acceptability was also assessed as a global hedonic response.

The intensity of each descriptive attribute was rated using a structured nine-point category scale, ranging from 1 (not perceptible or extremely low intensity) to 9 (extremely high intensity). The sensory terminology and assessment procedure were adapted from previously reported methods for olive-based products and table olives [2,7,28]. For each attribute, median scores were calculated and used to generate radar plots describing the sensory profiles of the formulations before and after storage.

### 2.7. Statistical Analysis

Data were expressed as mean ± standard deviation of three independent replicates (*n* = 3) for each formulation, storage temperature, and sampling time.

Multifactorial analysis of variance (ANOVA) was applied to evaluate the main effects of formulation, temperature, and storage time, as well as their interactions, on the main quality parameters of olive pâté during storage. When significant differences were detected, Tukey’s post hoc test was used for multiple comparisons. Differences were considered statistically significant at *p* ≤ 0.05.

Principal component analysis (PCA) was performed as an exploratory multivariate approach to obtain an integrated representation of the main physicochemical, colour, and antioxidant descriptors of olive pâté during storage. The analysis was carried out on the mean values corresponding to each experimental condition. The variables included in the analysis were pH, TPC, DPPH radical scavenging activity, and the CIELab colour coordinates (L, a, and b*). Before PCA, all variables were autoscaled to unit variance in order to account for differences in measurement units and magnitude. The first two principal components were retained for graphical interpretation, and sample scores and variable loadings were jointly represented in a biplot.

All statistical analyses were performed using IBM SPSS Statistics for Windows, Version 29.0 (IBM Corp., Armonk, NY, USA).

## 3. Results and Discussion

### 3.1. Technological and Functional Characterization of Microencapsulated Bergamot Extract (MBE)

**Figure 2:**
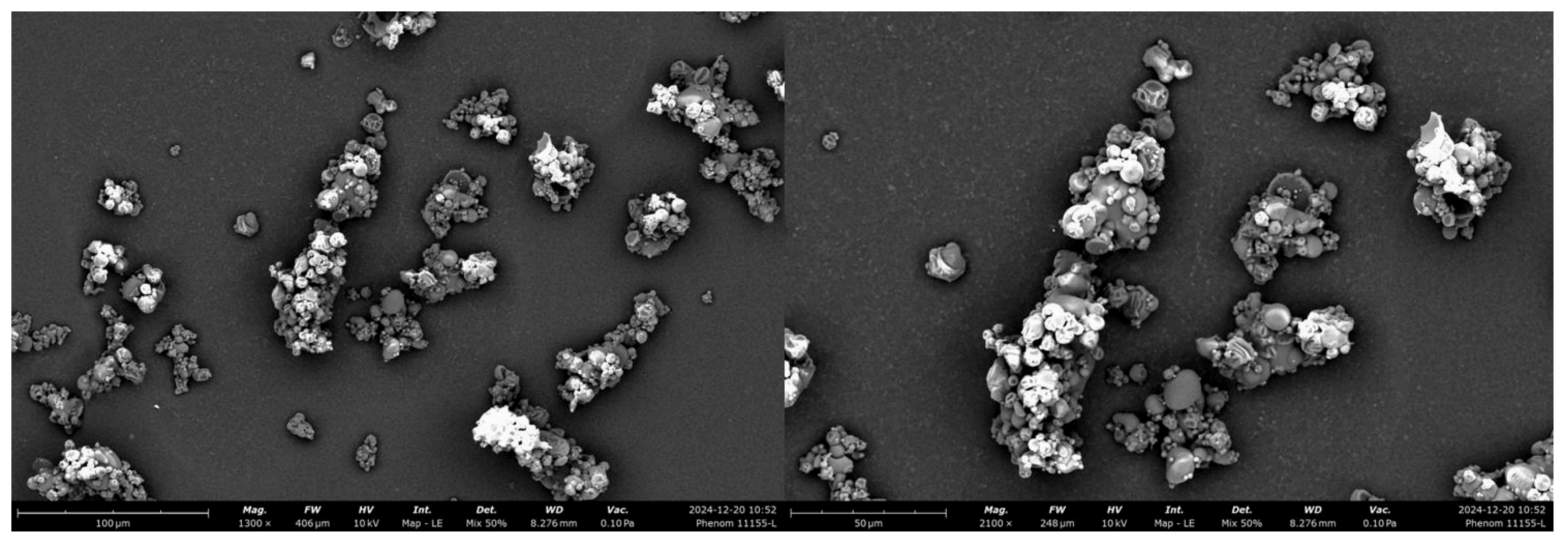
SEM micrographs of MBE obtained by spray drying.

The physicochemical, antioxidant, phenolic, and morphological characteristics of MBE are reported in Table 2 and Figure 2. The microencapsulated powder showed a moisture content of 4.96% and a water activity (a_w_) of 0.26, values consistent with those generally reported for stable food powders produced by spray drying [22,29,30]. a_w_ values below 0.30 are generally associated with reduced molecular mobility, limited microbial growth, and improved storage stability of dehydrated food ingredients. The low residual moisture content and a_w_ indicate suitable physicochemical conditions for preserving the integrity of the encapsulated extract before its incorporation into olive pâté.

MBE was characterized by a total phenolic content of 7.13 ± 0.07 mg GAE g^−1^ DM and antioxidant activity of 11.04 ± 0.02 and 3.11 ± 0.09 mmol TE g^−1^ DM, as determined by the ABTS and DPPH assays, respectively (Table 2). Differences between the two assay responses likely reflect their distinct radical systems and analytical conditions. Accordingly, ABTS and DPPH values were interpreted as complementary indicators of antioxidant activity rather than directly comparable measures.

UHPLC–PDA analysis further characterized the phenolic profile of MBE, confirming the presence of the main flavanones typically associated with bergamot, namely neoeriocitrin, naringin, neohesperidin, melitidin, and brutieridin. Among the quantified compounds, naringin was the most abundant (5.24 ± 0.02 mg g^−1^ DM), followed by neoeriocitrin, neohesperidin, brutieridin and melitidin. The detection of melitidin and brutieridin, two 3-hydroxy-3-methylglutaryl (HMG)-substituted flavanones considered characteristic of *Citrus bergamia*, supports the botanical origin of the extract and indicates that these compounds remained detectable after extraction and spray drying. The phenolic profile was consistent with that previously reported for bergamot by-products and microencapsulated bergamot extracts [11].

The spectrophotometric and chromatographic analyses provided complementary information regarding the composition of MBE. While the Folin–Ciocalteu assay estimates the overall reducing capacity of the extractable phenolic fraction, expressed as gallic acid equivalents, UHPLC–PDA specifically identifies and quantifies individual phenolic compounds. Consequently, the two analytical approaches should be regarded as complementary rather than directly comparable.

Based on the formulation composition (Table 1), the theoretical MBE-derived phenolic contributions were estimated at 16.94 and 33.88 mg GAE per 100 g of olives for OP_2.5_ and OP_5_, respectively.

Representative SEM micrographs of the spray-dried powder are shown in Figure 2. The microcapsules appeared predominantly spherical to sub-spherical and exhibited a marked tendency to form agglomerates. Several particles showed surface depressions and concavities, morphological features commonly observed in maltodextrin-based spray-dried systems as a consequence of rapid solvent evaporation, crust formation, and particle shrinkage during drying [31,32,33,34]. Importantly, no evidence of extensive particle collapse or severe structural disruption was observed, suggesting that maltodextrin provided an adequate encapsulating matrix capable of protecting the phenolic extract during the drying process.

The technological characterization demonstrated that MBE possessed suitable physicochemical stability, measurable antioxidant activity, and a phenolic profile characteristic of bergamot by-products, supporting its application as a functional ingredient in olive pâté. Nevertheless, the effectiveness of encapsulated antioxidants cannot be inferred solely from the properties of the powder itself. Once incorporated into a complex lipid-rich food matrix, their technological performance is expected to depend on several interacting factors, including the release of phenolic compounds from the maltodextrin matrix, their interactions with endogenous olive phenolics, and the storage conditions [16]. Consequently, the behaviour of the enriched formulations was subsequently evaluated throughout storage to determine how the level of MBE enrichment influenced product stability and quality preservation during storage.

### 3.2. Storage Stability of Olive Pâté Formulations

The effect of MBE incorporation on the storage stability of olive pâté was evaluated using two enrichment levels, corresponding to 2.5 g (OP_2.5_) and 5 g (OP_5_) of MBE per 100 g of olives, together with a non-enriched control (CTR). The two MBE concentrations were selected to investigate whether the response of the olive pâté matrix was influenced by the level of enrichment and to identify a formulation capable of providing a balanced stabilizing effect during storage.

The formulations were stored at 20 and 30 °C for 100 days. The two storage temperatures were selected to evaluate the stability behaviour of the formulations under different thermal conditions and to investigate whether exposure to a moderately elevated temperature affected the evolution of the main quality parameters during storage.

#### 3.2.1. Colour Stability and Chromatic Evolution During Storage

**Figure 3:**
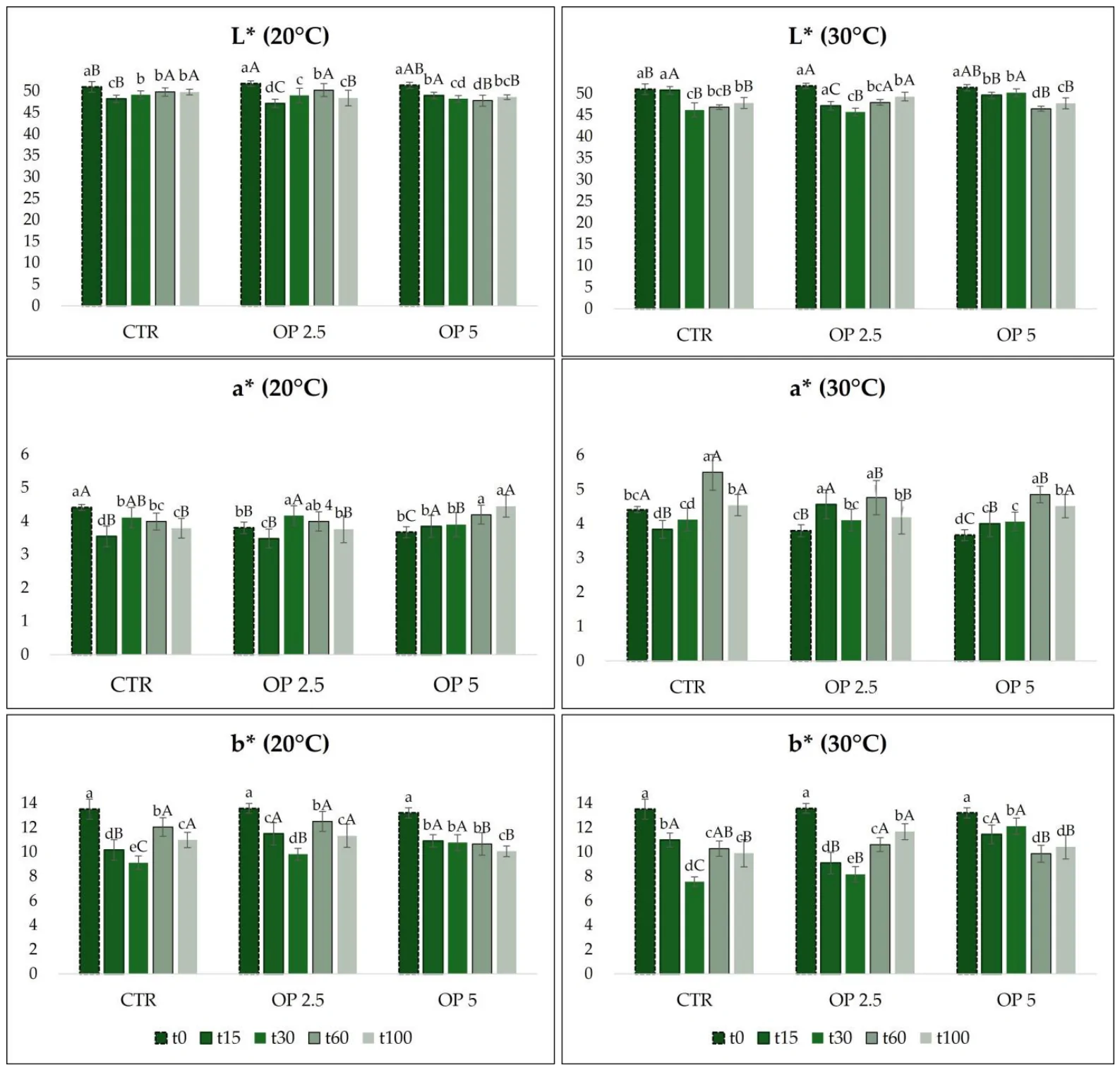
Evolution of colour parameters (L*, a*, and b*) in olive pâté formulations during storage at 20 and 30 °C. Different lowercase letters indicate significant differences among storage times within the same formulation, whereas different uppercase letters indicate significant differences among formulations at the same storage time, according to Tukey’s HSD test (*p* ≤ 0.05).

**Figure 4:**
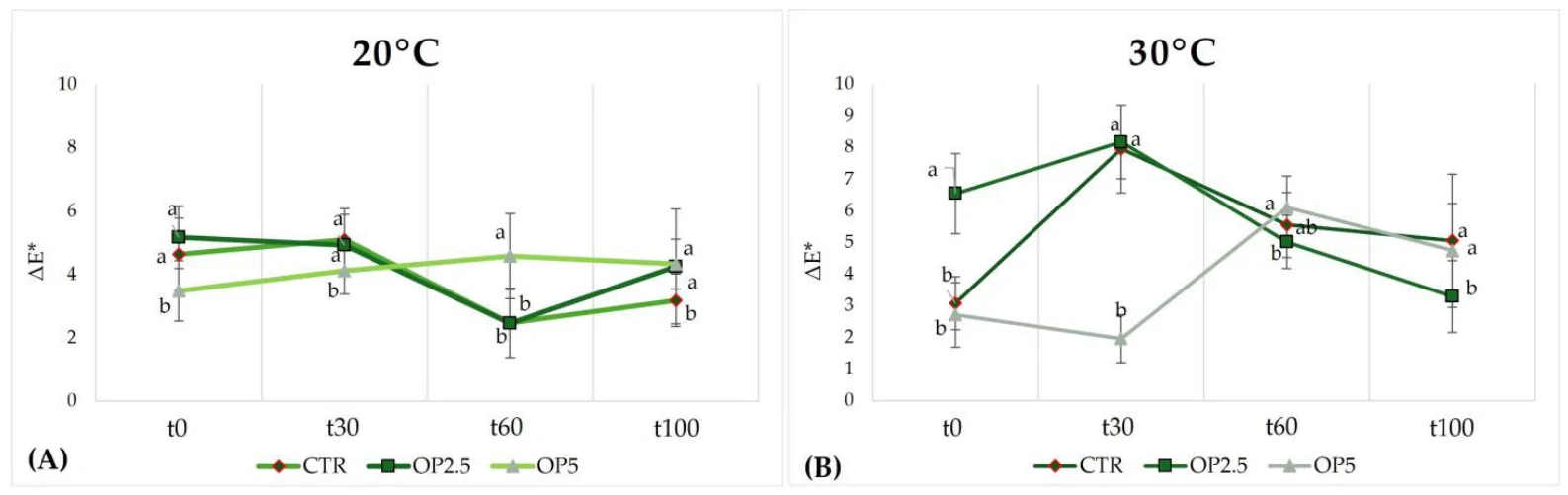
Temporal evolution of total colour difference (ΔE*) in olive pâté formulations during storage at (**A**) 20 °C and (**B**) 30 °C. ΔE values were calculated relative to the corresponding Day 0 colour coordinates. Data are expressed as mean ± standard deviation. Different lowercase letters indicate significant differences among formulations at the same storage time according to Tukey’s HSD test (*p* ≤ 0.05).

Colour stability is one of the main quality attributes influencing the commercial acceptance of olive pâté, as consumers generally associate colour preservation with freshness and product quality. The evolution of the CIELab colour coordinates (L*, a*, and b*) during storage is reported in Figure 3, while the temporal evolution of total colour difference (ΔE*) is shown in Figure 4.

In general, storage induced formulation-dependent chromatic modifications under both temperature conditions. However, colour evolution did not follow a uniform or progressively deteriorating pattern, indicating that the response of the olive pâté matrix was governed by the interaction between storage time, temperature, and MBE enrichment level rather than by temperature alone.

Among the evaluated colour descriptors, the total colour difference (Figure 4) provided the clearest overall assessment of formulation performance, as it integrates the combined variations in L*, a*, and b*. The temporal evolution of ΔE* showed a non-monotonic pattern, with formulation-dependent differences emerging at different stages of storage. At 20 °C, OP_5_ showed the lowest ΔE* values during the early storage period, whereas at day 60 it exhibited the highest colour difference. At 30 °C, OP_2.5_ showed relatively high ΔE* values at days 15 and 30, followed by a progressive decrease during the later stages of storage, while OP_5_ showed the opposite tendency, with lower initial values and a marked increase at day 60. After 100 days at 20 °C, the control formulation (CTR) exhibited the lowest colour variation (ΔE* = 3.18 ± 0.83), whereas OP_2.5_ and OP_5_ reached values of 4.25 ± 1.81 and 4.33 ± 0.79, respectively (Figure 4). Therefore, under standard storage conditions, MBE enrichment did not consistently reduce the overall chromatic deviation under storage at 20 °C. A different behaviour was observed at 30 °C, where OP_2.5_ showed the lowest ΔE* value (3.29 ± 1.13), significantly lower than CTR (5.05 ± 2.10) and OP_5_ (4.74 ± 1.49), whereas no significant difference was observed between CTR and OP_5_. Colour differences are generally considered clearly perceptible to an untrained observer when ΔE* values reach or exceed approximately 5 [35], and CTR was the only formulation to exceed this threshold after storage at 30 °C. These findings indicate that the protective effect of MBE on colour stability depended not only on its presence but also on the enrichment level and storage temperature. The intermediate enrichment level provided the best colour preservation under the most challenging storage condition, whereas doubling the amount of encapsulated extract did not confer additional long-term benefits.

The individual CIELab coordinates provide further insight into the mechanisms underlying these overall colour changes. Lightness (L*) showed a non-monotonic evolution throughout storage, with temporary decreases followed by partial recovery depending on formulation and storage condition (Figure 3). Although reductions in L* were observed in all formulations for each temperature, the subsequent evolution differed considerably among treatments, suggesting that colour development was influenced by formulation-specific interactions rather than by progressive darkening alone. Similar decreases in lightness have been reported for olive pâté and other olive-derived products during storage and have generally been associated with oxidative phenomena and pigment transformations [36,37]. However, the fluctuating behaviour observed in the present study suggests that multiple concurrent processes, including pigment degradation, redistribution within the matrix, and light scattering effects associated with structural changes, may contribute to the observed variations.

Changes in red–green coordinate (a*) were relatively limited at 20 °C, whereas a transient increase was observed at 30 °C, particularly during the intermediate storage period, followed by a partial decrease after prolonged storage. Similar increases in a* have previously been described during the storage of olive pâté [37] and have been related to progressive chlorophyll degradation and the formation of brownish pigments. Nevertheless, the absence of a consistent temporal trend in the present study suggests that changes in a* cannot be attributed to a single degradation pathway but rather reflect the simultaneous evolution of multiple pigment systems within the olive matrix.

Among the individual colour coordinates, b* showed the greatest ability to discriminate between formulations. A general reduction in yellowness occurred during storage, although the extent of this decrease depended on both storage temperature and enrichment level. At 30 °C, OP_5_ retained higher b* values during the early stages of storage, whereas OP_2.5_ exhibited superior preservation after 100 days, reaching the highest final b* value among the three formulations. This observation indicates that the initial advantage associated with the higher enrichment level was not maintained over prolonged storage. Similar reductions in b* have been associated with the degradation of carotenoid pigments and other colour-related compounds in processed olive products [38,39].

Taken together, the evolution of the individual CIELab coordinates and the integrated ΔE* values demonstrate that the effect of MBE on colour preservation depended on the enrichment level but was not proportional to the amount incorporated. The intermediate enrichment level (OP_2.5_) consistently provided the best compromise between colour preservation and storage stability under the most demanding thermal conditions, whereas the higher enrichment level failed to produce a proportional improvement. These findings highlight that, in complex lipid-rich matrices such as olive pâté, the effectiveness of encapsulated natural antioxidants cannot be explained solely by the amount incorporated. Instead, colour stability likely depends on the behaviour of encapsulated phenolics within the olive matrix, together with endogenous olive constituents and the overall oxidative behaviour of the product. Further insight into these mechanisms is provided by the evolution of phenolic content, antioxidant activity, and oxidative stability discussed in the following sections.

#### 3.2.2. pH Stability During Storage

The evolution of pH during storage is reported in Table 3. Overall, pH remained remarkably stable throughout the 100-day storage period, irrespective of formulation or storage temperature, indicating that the incorporation of MBE did not substantially modify the acid–base balance of the olive pâté matrix.

The pH values ranged from 3.91 to 4.14, remaining consistently below 4.5 throughout storage. Although statistically significant differences were observed at specific sampling times, no consistent temporal trend or enrichment-level-dependent effect was evident. The maintenance of an acidic environment is technologically relevant because it contributes to microbiological stability and limits several degradation reactions affecting olive-based products during storage. Similar pH values have previously been reported for pasteurized olive pâtés and fermented olive products [36,37]. Moreover, the absence of marked pH variations indicates that the colour modifications discussed in the previous Section 3.2.1 are unlikely to be associated with acidification phenomena, but rather with oxidative and pigment-related processes occurring during storage.

The overall stability of pH demonstrates that the olive pâté matrix remained physiochemically stable throughout storage. Consequently, the differences observed among formulations in colour evolution, phenolic composition, antioxidant activity, and oxidative stability cannot be attributed to substantial changes in pH, but more likely reflect the different behaviour of the incorporated MBE within the olive matrix.

#### 3.2.3. Phenolic Content and Antioxidant Activity: Matrix-Dependent Response

**Figure 5:**
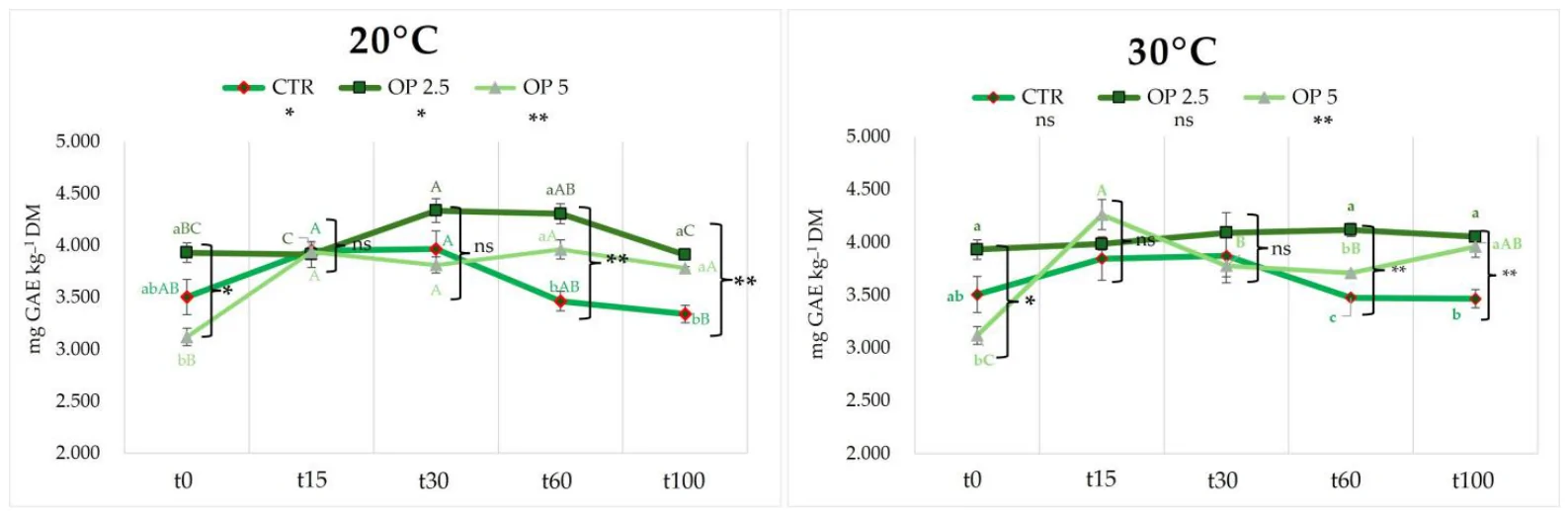
Evolution of TPC in olive pâté formulations during storage at 20 and 30 °C. Different uppercase letters indicate significant differences among storage times within the same formulation, whereas different lowercase letters indicate significant differences among formulations at the same storage time, according to Tukey’s post hoc test (*p* ≤ 0.05). Abbreviations: **, significance at *p* < 0.01; *, significance at *p* < 0.05; ns, not significant.

**Figure 6:**
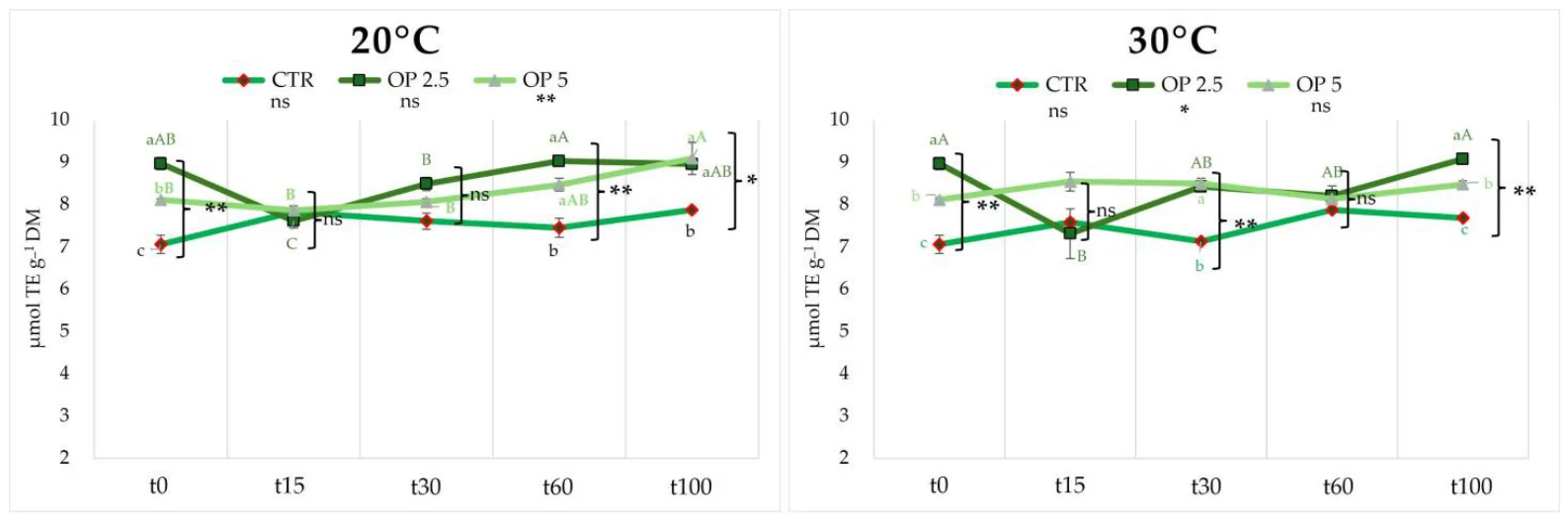
Evolution of DPPH radical scavenging activity in olive pâté formulations during storage at 20 and 30 °C. Different uppercase letters indicate significant differences among storage times within the same formulation, whereas different lowercase letters indicate significant differences among formulations at the same storage time, according to Tukey’s post hoc test (*p* ≤ 0.05). Abbreviations: **, significance at *p* < 0.01; *, significance at *p* < 0.05; ns, not significant.

Total phenolic content (TPC) and DPPH radical scavenging activity were monitored throughout storage to investigate the evolution of the antioxidant fraction of olive pâté and to assess the effect of MBE on the functional stability of the product (Figure 5 and Figure 6). Overall, both parameters exhibited formulation-dependent responses, although their temporal evolution was not always parallel, indicating that changes in the Folin-reactive fraction were not necessarily accompanied by corresponding changes in antioxidant activity.

TPC values ranged from approximately 3120 to 4470 mg GAE kg^−1^ DM (Figure 5). TPC did not show a progressive decline during storage but followed formulation- and temperature-dependent trajectories. At 20 °C, significant temporal variations were observed in all formulations. OP_2.5_ exhibited comparatively high TPC values throughout storage, whereas OP_5_ showed the largest temporal increase despite its lower initial phenolic content. At 30 °C, OP_2.5_ again maintained relatively stable phenolic levels, while significant temporal changes were detected only in OP_5_. These observations indicate that the response of the phenolic fraction was influenced by the enrichment level but did not increase proportionally with the amount of MBE incorporated.

The interpretation of TPC evolution requires consideration of the analytical characteristics of the Folin–Ciocalteu assay. Based on the measured dry matter and phenolic content of MBE, the theoretical phenolic contribution introduced into the formulations was only 16.94 and 33.88 mg GAE per 100 g of olives for OP_2.5_ and OP_5_, respectively (Table 1). These values represent the theoretical phenolic input derived exclusively from the added MBE and provide a reference for interpreting the TPC measured in the enriched pâté. However, the phenolic content measured in the olive pâté was substantially higher than the theoretical contribution of MBE alone, indicating that the observed TPC values were largely determined by the endogenous phenolic fraction of the olive matrix. Their evolution during storage may also have been influenced by changes in phenolic extractability and by interactions among olive- and bergamot-derived compounds. Therefore, the temporal evolution of TPC cannot be explained simply by the quantitative contribution of bergamot-derived phenolics or by their gradual release from the microcapsules. Rather, it likely reflects changes in the extractability of endogenous olive phenolics together with interactions between olive- and bergamot-derived compounds during storage. Similar formulation-dependent changes have been reported for enriched olive-based products containing encapsulated natural extracts [15,35].

Although TPC provides useful information on the evolution of the extractable reducing fraction, it does not necessarily reflect the antioxidant effectiveness of the system. This aspect became evident when TPC was compared with DPPH radical scavenging activity (Figure 6). DPPH values ranged from approximately 7.0 to 9.1 μmol TE g^−1^ DM and showed formulation-dependent variations that only partially mirrored the evolution of TPC.

At the beginning of storage, both enriched formulations exhibited higher radical scavenging activity than the control, confirming the functional contribution of MBE immediately after incorporation into the olive matrix. During storage at 20 °C, the initial differences among formulations became less pronounced, although both OP_2.5_ and OP_5_ maintained higher antioxidant activity than CTR during the later stages of storage. By day 100, OP_5_ reached the highest mean DPPH value, while OP_2.5_ showed comparable antioxidant performance. Under storage at 30 °C, the antioxidant response evolved differently. Although differences among formulations fluctuated throughout storage, OP_2.5_ exhibited the highest final DPPH activity after 100 days, followed by OP_5_ and the control formulation. Consequently, the intermediate enrichment level provided the highest antioxidant activity after prolonged storage at 30 °C.

The different trajectories observed for TPC and DPPH indicate that antioxidant functionality was not governed exclusively by the amount of Folin-reactive compounds present in the pâté. The antioxidant response of the system is more likely determined by qualitative changes in phenolic composition, differences in the reactivity of individual compounds, and their accessibility within the olive matrix. Similar discrepancies between total phenolic content and antioxidant activity have frequently been reported in complex plant-derived food systems, where phenolic composition and compound interactions often exert a greater influence on antioxidant behaviour than total phenolic concentration alone [40,41].

In conclusion, the combined evaluation of TPC and DPPH demonstrates that MBE influenced the antioxidant behaviour of olive pâté in a formulation-dependent manner. OP_2.5_ maintained comparatively stable phenolic levels throughout storage while providing the highest antioxidant activity after prolonged storage at 30 °C. In contrast, OP_5_ showed greater temporal variation in the Folin-reactive fraction without achieving a proportional improvement in antioxidant performance. These findings indicate that the technological effectiveness of MBE depended not simply on the amount incorporated but on its interaction with the olive matrix, further supporting the need to optimize the enrichment level rather than maximizing the concentration of added antioxidants.

Further insight into the behaviour of MBE during storage was provided by UHPLC analysis of individual phenolic compounds. The analysis confirmed the presence of the bergamot-derived phenolic compounds quantified in the original MBE, namely neoeriocitrin, naringin, and neohesperidin, in the enriched olive pâté formulations (Table 4). Only compounds previously quantified in MBE and consistently detected in the enriched pâté were reported. After 100 days of storage at both temperatures, these compounds remained detectable, with only limited quantitative variations depending on the storage condition.

The main endogenous olive phenolics, including hydroxytyrosol, tyrosol, and luteolin, were also identified in all formulations. Their concentrations showed only minor changes during storage and no consistent formulation-dependent trend, suggesting that MBE enrichment did not substantially affect the evolution of the native phenolic profile of the olive matrix.

The persistence of the characteristic bergamot flavanones throughout storage is consistent with the enhanced antioxidant behaviour observed in the enriched formulations, particularly the greater oxidative stability demonstrated by the Oxitest analysis. Although the theoretical phenolic contribution provided by MBE represented only a limited fraction of the total phenolic content of the product, the UHPLC results demonstrate that the added flavanones remained associated with the olive matrix during storage, suggesting their contribution to the enhanced oxidative resistance of the enriched formulations. Similar behaviour has been reported for other bergamot-derived ingredients, where characteristic flavanones remained detectable after food processing and storage, confirming the protective role of microencapsulation in preserving these bioactive compounds and facilitating their incorporation into different food matrices [16,42].

#### 3.2.4. Microbiological Stability During Storage

Microbiological stability is a key quality attribute of pasteurized olive pâté, particularly during prolonged storage under ambient conditions. The evolution of total aerobic bacteria counts (CBT) and yeasts during storage is reported in Table 5. No viable microorganisms were detected in freshly prepared samples, confirming the effectiveness of pasteurization and hygienic processing conditions. After 100 days of storage, microbial counts remained very low in all formulations, although differences among formulations became more evident, particularly at 30 °C. CBT counts were consistently lower in the MBE-enriched formulations than in the control. At 20 °C, bacterial growth was detected only in CTR, whereas no viable aerobic bacteria were detected in either OP_2.5_ or OP_5_. Storage at 30 °C resulted in a moderate increase in bacterial counts, particularly in the control formulation (0.74 ± 0.10 log CFU g^−1^), while OP_2.5_ and OP_5_ maintained significantly lower populations (0.30 ± 0.00 and 0.00 log CFU g^−1^, respectively). Although statistically significant, these differences were of limited practical relevance, as microbial counts remained consistently low throughout storage in all formulations.

Yeasts were the predominant microorganisms detected at the end of the storage period. Significant increases were observed in all formulations; however, both MBE-enriched pâtés consistently exhibited lower yeast counts than the control at both storage temperatures. This effect was particularly evident at 30 °C, where yeast populations decreased from 1.84 ± 0.10 log CFU g^−1^ in CTR to 1.52 ± 0.02 and 1.32 ± 0.02 log CFU g^−1^ in OP_2.5_ and OP_5_, respectively. Moulds were not detected in any formulation throughout the experimental period. These results reflect microbiological stability rather than a comprehensive safety assessment, as specific pathogens and lactic acid bacteria were not evaluated.

The overall microbiological stability observed in the present study is consistent with the hurdle technology concept, whereby multiple preservation factors, including acidic pH, pasteurization and hermetic packaging, act synergistically to inhibit microbial proliferation [43]. Similar microbiological behaviour has been reported for olive pâté and related olive-based products stored under acidic conditions [37,44].

The lower bacterial and yeast counts observed in the MBE-enriched formulations suggest that the phenolic-rich ingredient may have contributed to microbial control. However, because no specific antimicrobial or challenge tests were performed, these findings should not be interpreted as direct evidence of antimicrobial activity. Nevertheless, the observed trend is consistent with previous studies reporting reduced microbial populations in olive pâté enriched with phenolic-rich plant extracts. Difonzo et al. [37] reported reductions of approximately 0.5–1 log cycle in the main microbial groups of olive pâté enriched with olive leaf extract, particularly at the higher enrichment level, whereas Cosmai et al. [36] observed greater microbial inhibition with increasing concentrations of a natural *Allium* spp. extract.

At the end of storage, the MBE-enriched pâtés showed a more favorable microbiological profile than the control, with OP5 exhibiting the lowest total aerobic bacterial and yeast counts, particularly at 30 °C. This response differed from the color and antioxidant results, for which OP_2.5_ generally showed the most balanced behavior, indicating that the technological performance of MBE depended on the quality parameter considered.

In conclusion, MBE enrichment contributed to maintaining low microbial populations throughout storage. Microbiological stability tended to improve with increasing MBE concentration, with OP_5_ providing the lowest final counts. These findings further demonstrate that the technological performance of MBE cannot be adequately assessed using a single quality attribute and highlight the importance of optimizing the enrichment level according to the desired balance among physicochemical, antioxidant, sensory, and microbiological properties.

#### 3.2.5. Oxidative Stability During Storage

Oxidative stability is a key quality attribute of lipid-rich foods because it directly affects shelf life, flavour preservation, nutritional quality, and consumer acceptance. In the present study, oxidative stability was evaluated using the Oxitest method and expressed as the induction period (IP), with longer induction periods indicating greater resistance to accelerated lipid oxidation [26,45].

**Figure 7:**
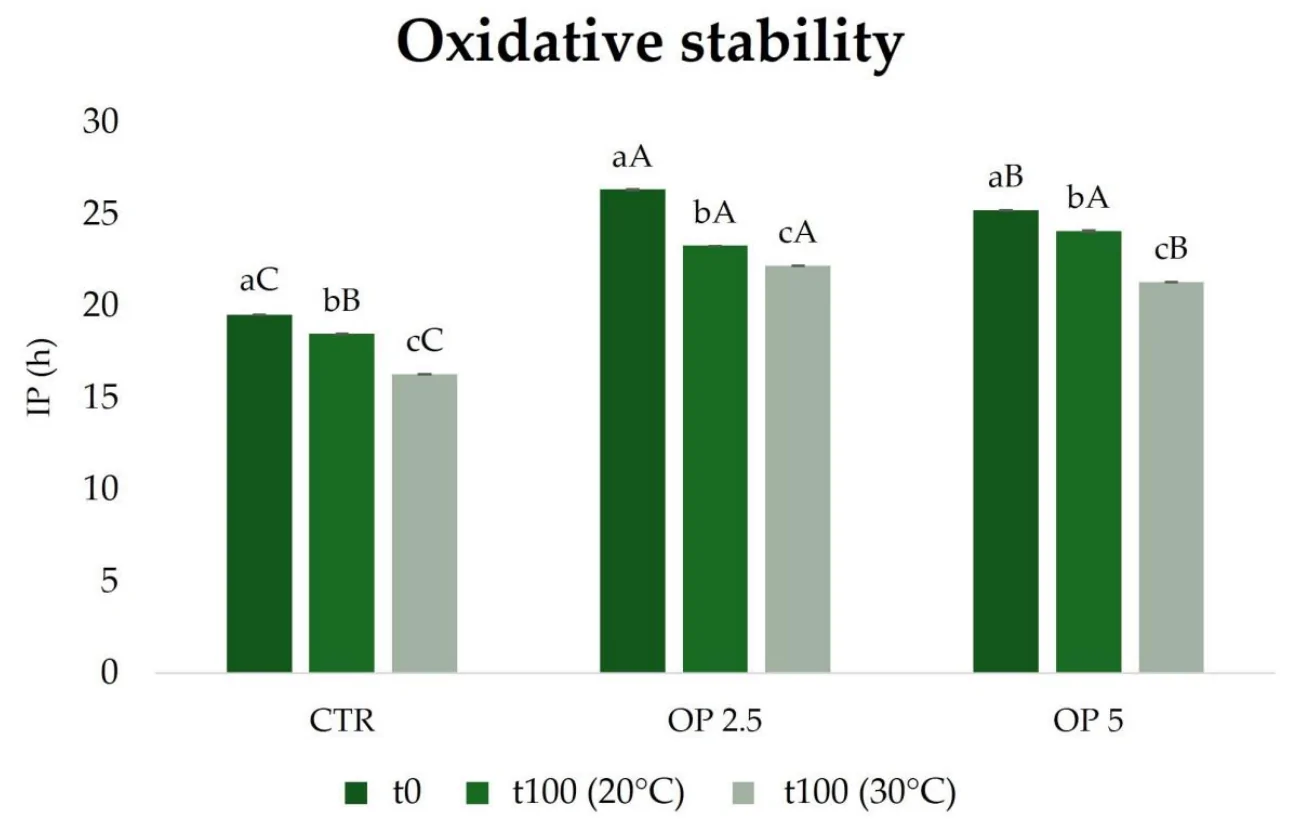
Induction period (IP) of olive pâté formulations at the beginning and after 100 days of storage at 20 and 30 °C. Different lowercase letters indicate significant differences among storage times within the same formulation, whereas different uppercase letters indicate significant differences among formulations at the same storage time, according to Tukey’s HSD test (*p* ≤ 0.05).

MBE incorporation increased the oxidative stability of olive pâté from t0 (Figure 7). The control formulation showed an initial IP of 19.51 h, whereas OP_2.5_ and OP_5_ reached 26.32 and 25.20 h, respectively. Compared with CTR, these values corresponded to increases of approximately 35% for OP_2.5_ and 29% for OP_5_. However, increasing the enrichment level from 2.5 to 5.0 g MBE per 100 g of olives did not produce a further increase in the initial IP, indicating that oxidative protection was influenced by the enrichment level but was not proportional to the amount incorporated.

After 100 days of storage at 20 °C, the IP decreased to 18.47 h in CTR, 23.26 h in OP_2.5_, and 24.07 h in OP_5_. Under these conditions, both enriched formulations maintained greater oxidative stability than the control, with OP_5_ showing the highest final IP. A more pronounced reduction was observed after storage at 30 °C, with IP values of 16.27, 22.18, and 21.30 h for CTR, OP_2.5_, and OP_5_, respectively. Nevertheless, both MBE-enriched formulations retained substantially greater resistance to oxidation than CTR. Notably, the IP of OP_2.5_ after 100 days at 30 °C remained higher than the initial value recorded for the control formulation (22.18 versus 19.51 h).

The response of the enriched formulations therefore varied according to storage temperature. OP_2.5_ exhibited the highest initial oxidative stability and retained the longest induction period after storage at 30 °C, whereas OP_5_ showed a slight advantage after 100 days at 20 °C. These findings confirm that increasing the MBE level did not result in a uniform or proportional improvement in oxidative protection. Instead, the relative performance of the two enriched formulations depended on the storage condition considered.

The Oxitest results are also consistent with the antioxidant behaviour discussed in the previous Section 3.2.3. OP_2.5_ combined relatively stable total phenolic content with the highest DPPH radical scavenging activity after storage at 30 °C and the longest induction period, whereas OP_5_ exhibited greater temporal changes in the Folin-reactive fraction without achieving a proportional increase in oxidative stability. This confirms that the antioxidant effectiveness of the formulations cannot be explained solely by the total concentration of phenolic compounds, but rather depends on their chemical composition, accessibility, and interactions within the food matrix.

The longer induction periods observed for OP_2.5_ and OP_5_ may instead reflect interactions between bergamot-derived flavanones, endogenous olive antioxidants, and the lipid phase. Such interactions could influence the initiation and propagation of oxidative reactions and thereby improve the overall resistance of the product to oxidation.

Overall, Oxitest analysis demonstrated that MBE enrichment improved the oxidative stability of olive pâté during storage. However, increasing the enrichment level from 2.5 to 5.0 g per 100 g of olives did not provide consistently greater protection. OP_2.5_ showed the most favourable performance under the more demanding storage condition of 30 °C, whereas OP_5_ retained a slight advantage at 20 °C. These results reinforce the need to define the optimal enrichment level by considering the overall quality profile of the product rather than maximizing the amount of antioxidant ingredient added.

#### 3.2.6. Sensory Evaluation

**Figure 8:**
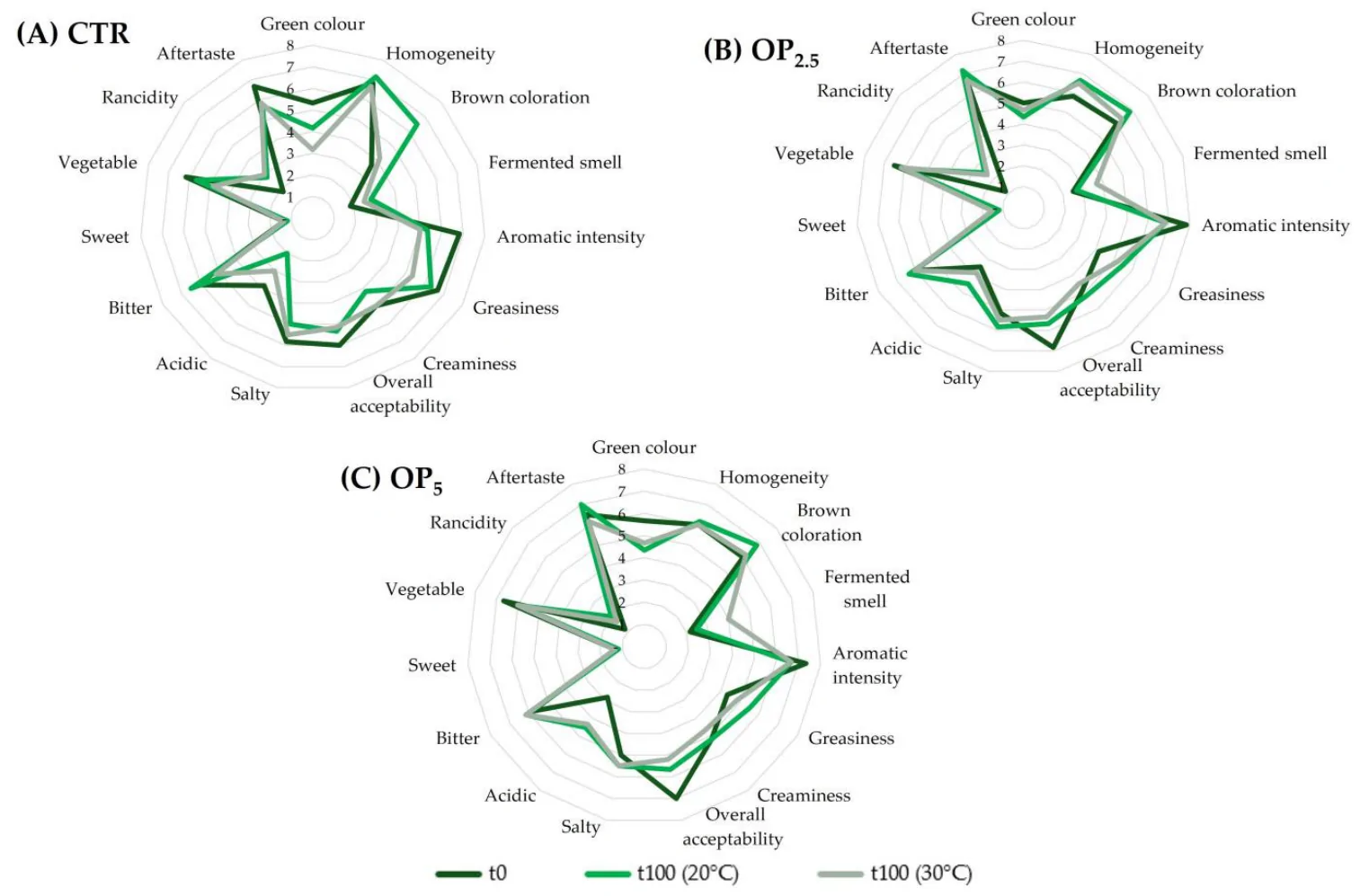
Sensory profiles of olive pâté formulations before storage and after 100 days of storage at 20 and 30 °C.

The sensory profiles of the olive pâté formulations at t0 and after 100 days of storage at 20 and 30 °C are presented in Figure 8. Overall, storage induced moderate changes in the sensory profiles of the formulations, while the incorporation of microencapsulated bergamot extract (MBE) did not adversely affect the typical sensory characteristics of olive pâté. Radar plots were used to provide an integrated visual comparison of the formulations rather than to emphasize differences in individual descriptors. Similar observations have been reported for olive-based pâtés enriched with natural plant extracts, in which antioxidant ingredients improved product stability without generating undesirable sensory defects [37].

The visual descriptors showed the most evident storage-related changes. Green colour generally decreased during storage, particularly at 30 °C, whereas brown colouration tended to increase in all formulations, reflecting the evolution of pigment-related phenomena. These changes appeared less pronounced in OP_2.5_ than in CTR. This observation was consistent with the instrumental colour measurements, as OP_2.5_ showed the lowest ΔE* value after 100 days at 30 °C, indicating better preservation of the original colour under the most demanding storage condition. The correspondence between sensory perception and instrumental colour analysis supports the reliability of the observed colour trends and suggests that the intermediate enrichment level more effectively preserved the visual quality of olive pâté.

Aromatic intensity remained generally well preserved throughout storage, whereas fermented smell showed only limited variations among formulations. The absence of evident fermentative off-odours was consistent with the microbiological results, which showed very low bacterial and yeast populations throughout storage. The sensory findings therefore support the effectiveness of the combined preservation factors, including pasteurization, acidic pH, and hermetic packaging, in maintaining product quality without substantially compromising its aromatic profile. Similar relationships between microbiological stability and sensory quality preservation have previously been reported for olive-based pâtés [37].

Among the taste-related descriptors, rancidity showed the most relevant formulation-dependent behaviour. Although the perceived differences were moderate, the MBE-enriched formulations, particularly OP_2.5_, tended to show lower rancidity scores than CTR after storage. This trend was consistent with the Oxitest results, which indicated greater oxidative resistance in the enriched formulations, and with the higher antioxidant activity retained by OP_2.5_ after prolonged storage. The correspondence between instrumental and sensory data suggests that improved oxidative stability contributed to limiting the development of oxidation-related sensory defects.

The remaining taste-related descriptors, including bitterness, acidity, sweetness, and vegetable notes, showed only minor variations throughout storage, indicating that MBE incorporation did not substantially modify the characteristic flavour profile of the pâté. Aftertaste also remained relatively stable, suggesting that the addition of the phenolic-rich microencapsulated extract did not generate marked undesirable residual sensations despite the presence of citrus-derived flavanones. The selection and interpretation of these descriptors were consistent with the sensory lexicon proposed for olive pâté by Lanza et al. [7].

Texture-related descriptors, including creaminess and greasiness, remained relatively stable regardless of formulation or storage temperature. This behaviour was consistent with the overall physicochemical stability observed during storage and suggests that the structural characteristics of the pâté matrix were largely preserved. The absence of substantial textural changes further indicates that MBE incorporation did not adversely affect the sensory perception of the product consistency. Similar findings have been reported for olive pâtés produced under different processing conditions or enriched with natural functional ingredients [10].

Overall quality remained high in all formulations throughout storage, indicating satisfactory preservation of the global sensory characteristics of the products. Among the tested formulations, OP_2.5_ showed the most balanced overall performance when the sensory findings were considered together with the colour, antioxidant, and oxidative stability results. The convergence between instrumental and sensory observations supports the conclusion that the technological effectiveness of MBE cannot be explained simply by increasing the enrichment level. Rather, the overall quality of olive pâté depended on achieving an appropriate balance between oxidative protection, colour preservation, and maintenance of its characteristic sensory attributes. These results support the selection of OP_2.5_ as the most suitable formulation among those investigated.

#### 3.2.7. Multivariate Analysis of Quality Parameters

**Figure 9:**
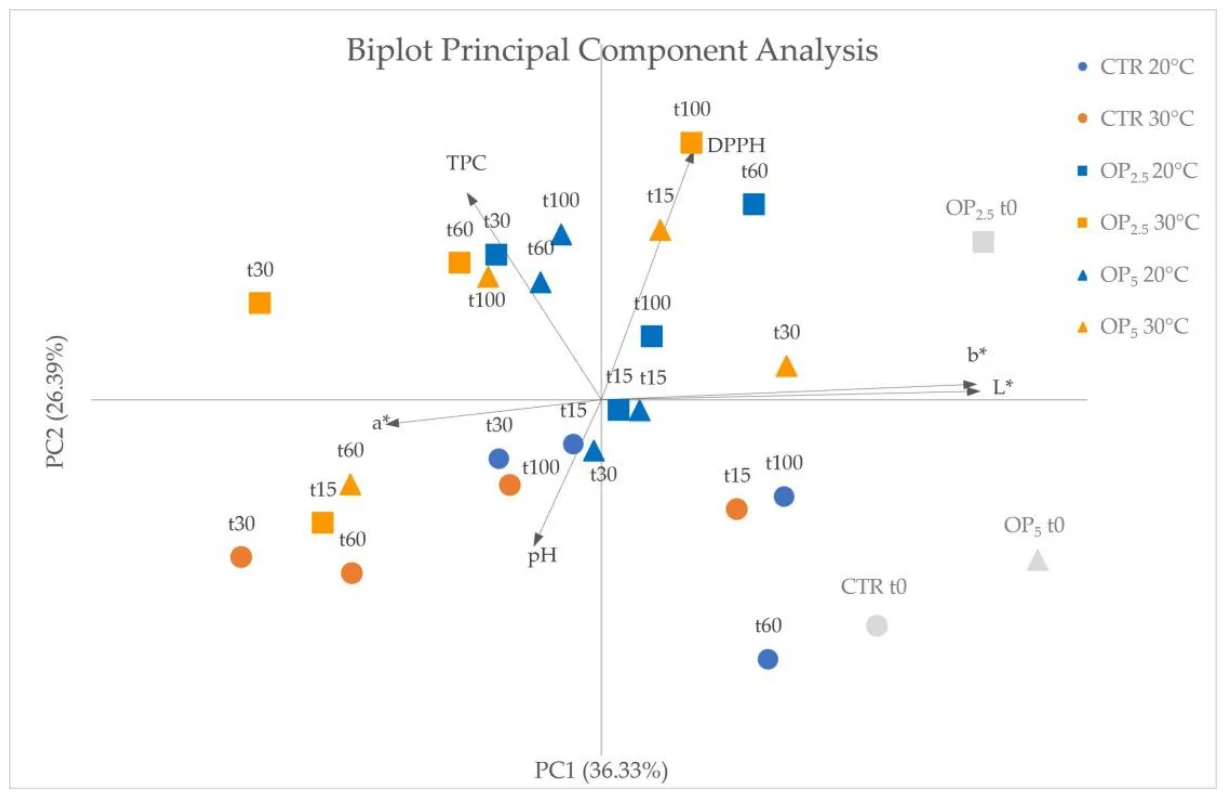
PCA score-loading biplot of olive pâté formulations based on physicochemical, colour, and antioxidant parameters during storage at 20 and 30 °C.

Principal component analysis was applied to provide an integrated interpretation of the main quality descriptors measured during storage, including physicochemical parameters, colour coordinates, and antioxidant-related variables. The first two principal components explained 62.72% of the total variance, with PC1 accounting for 36.33% and PC2 for 26.39% (Figure 9).

The loading plot indicated that PC1 was mainly associated with the colour coordinates L* and b*, which showed strong positive loadings, whereas a* was positioned in the opposite direction. The nearly parallel orientation of the L* and b* vectors indicates that lightness and yellowness varied coherently across the experimental conditions. Conversely, their opposition to a* suggests that samples with negative PC1 scores were comparatively associated with lower lightness and yellowness and with a greater contribution of the red–brown colour component. Therefore, PC1 represented the main chromatic gradient of the dataset. In contrast, PC2 was mainly influenced by antioxidant-related variables, particularly DPPH radical scavenging activity and TPC, indicating that this component reflected changes in the phenolic and antioxidant response of the matrix.

On the opposite side of DPPH and TPC, pH was negatively associated with PC2.

The score plot showed that the pre-storage samples were separated from several stored samples, confirming that storage induced a measurable shift in the overall quality profile. The three samples analysed at day 0 were located in the positive RC1 region and were clearly separated from most stored samples, indicating that storage modified the combined chromatic and antioxidant characteristics of the pâté.

The distribution of the stored samples also reflected the combined influence of formulation, storage temperature, and time. Relatively limited multivariate differentiation was observed at early storage. The separation became progressively more evident as time passed, although the trajectories appear evidently influenced by both formulation and temperature. This distribution did not follow a simple progression from CTR to OP_2.5_ and OP_5_, confirming that the response to MBE enrichment was not linearly dose-dependent.

At 20 °C, OP_2.5_ samples in the late storage times were positioned in the positive PC2 region associated with DPPH and TPC. OP_5_ samples at days 60 and 100 showed a similar positioning, whereas CTR samples at 60 and 100 days remained in the negative PC2 region, indicating a comparatively weaker association with antioxidant-related variables. OP5, however, showed a less linear trajectory, as the sample at t100 moved towards the antioxidant-associated region.

This variability indicates that the higher level of enrichment did not generate a uniformly higher multivariate profile. The effect of conservation at 30 °C was particularly evident for the CTR by placing it in the negative PC2 half-plane. A simultaneous change related to a lower phenolic concentration and the opposite direction to the L* and b* chromaticisms is evident. Sample OP_2.5_ at the beginning of storage (t15) shifted towards negative values of PC1 and PC2, to subsequently orient towards the positive region of PC2. In particular, at 30 °C, it positioned itself close to the DPPH vector, in line with the high radical activity observed.

OP_5_ samples stored at 30 °C were generally positioned in the positive PC2 region, but with a rather variable distribution, demonstrating that the increase in added MBE did not produce a stable or proportional improvement in the qualitative profile analyzed.

The preferential distribution of OP_2.5_ samples in the positive PC2 region during the later stages of storage, particularly at 30 °C, supports the favourable antioxidant behaviour of the intermediate enrichment level.

The PCA therefore validated the absence of a proportional relationship to the concentration of MBE added to the pâté formulation on antioxidant and colour preservation responses.

## 4. Conclusions

This study demonstrated that microencapsulated bergamot by-product extract is a promising clean-label ingredient for improving the storage stability of olive pâté produced from the Nocellara Messinese cultivar. The results showed that its effectiveness depended on the quality parameter considered and was not proportional to the amount incorporated, indicating that increasing the concentration of a natural antioxidant does not necessarily result in greater technological benefits.

Among the tested formulations, the intermediate enrichment level (OP_2.5_) provided the most balanced overall quality profile. It showed particularly favourable performance under storage conditions of 30 °C, with improved colour preservation, antioxidant activity, and oxidative stability, while maintaining the characteristic sensory properties of olive pâté. In contrast, at 20 °C, the effects of MBE enrichment were more dependent on the specific quality parameter considered. Although OP_5_ showed the lowest microbial counts at the end of storage, the higher enrichment level did not consistently provide additional advantages over OP_2.5_ for the other quality attributes evaluated.

The combined instrumental, microbiological, chromatographic, and sensory findings support the technological suitability of MBE within the olive pâté matrix. The persistence of bergamot-derived flavanones during storage, together with the endogenous phenolic fraction of the olives, suggests that the formulation-dependent response may reflect the combined influence of phenolic composition, compound accessibility, and the physicochemical characteristics of the food matrix. However, specific molecular interactions between bergamot- and olive-derived phenolics were not investigated and therefore cannot be inferred from the present results.

Overall, the present study demonstrates that optimizing the enrichment level is more important than maximizing the concentration of the antioxidant ingredient. These findings provide a scientific basis for the use of microencapsulated bergamot by-product extract as a functional ingredient in olive-based spreads while simultaneously promoting the valorisation of citrus processing by-products within a circular economy framework. Future research should investigate the behaviour of individual phenolic compounds during storage, validate these findings under industrial production conditions, and assess consumer acceptance in larger sensory studies.

## Figures and Tables

**Table 1:** Composition of the experimental olive pâté formulations per 100 g of olives.

Formulation	Olives (g)	Sunflower Oil (mL)	MBE (g)
**CTR**	100	10	0
**OP_2.5_**	100	10	2.5
**OP_5_**	100	10	5.0

**Table 2:** Physicochemical, antioxidant and phenolic characterization of MBE.

Parameters	Value
Moisture content (%)	4.96 ± 0.30
Water activity (a_w_)	0.26 ± 0.01
TPC (mg GAE g^−1^ DM)	7.13 ± 0.07
ABTS (mmol TE g^−1^ DM)	11.04 ± 0.02
DPPH (mmol TE g^−1^ DM)	3.11 ± 0.09
Neoeriocitrin (mg g^−1^ DM)	4.57 ± 0.03
Naringin (mg g^−1^ DM)	5.24 ± 0.02
Neohesperidin (mg g^−1^ DM)	4.30 ± 0.01
Melitidin (mg g^−1^ DM)	0.67 ± 0.01
Brutieridin (mg g^−1^ DM)	2.26 ± 0.02

**Table 3:** Evolution of pH in olive pâté formulations during storage at 20 and 30 °C.

	pH						
Samples	T	t0	t15	t30	t60	t100	Sign.
**CTR**	20 °C	4.03 ± 0.01 ^BC^	4.08 ± 0.02 ^AB^	4.06 ± 0.03 ^AB^	4.14 ± 0.01 ^A^	3.96 ± 0.03 ^C^	**
	30 °C	4.03 ± 0.01 ^AB^	4.08 ± 0.01 ^A^	4.09 ± 0.04 ^A^	4.02 ± 0.01 ^AB^	3.95 ± 0.03 ^B^	*
	Sign.	ns	ns	ns	**	ns	
**OP_2.5_**	20 °C	3.98 ± 0.02 ^D^	3.99 ± 0.01 ^CD^	4.05 ± 0.02 ^BC^	4.06 ± 0.01 ^AB^	4.11 ± 0.01 ^A^	**
	30 °C	3.98 ± 0.02 ^BC^	4.10 ± 0.01 ^A^	4.03 ± 0.01 ^AB^	3.94 ± 0.03 ^C^	3.91 ± 0.01 ^C^	**
	Sign.	ns	**	ns	*	**	
**OP_5_**	20 °C	4.01 ± 0.01 ^AB^	4.03 ± 0.04 ^AB^	4.08 ± 0.02 ^A^	3.97 ± 0.04 ^AB^	3.95 ± 0.02 ^B^	*
	30 °C	4.01 ± 0.01 ^B^	4.00 ± 0.01 ^BC^	4.02 ± 0.01 ^B^	4.10 ± 0.01 ^A^	3.96 ± 0.01 ^C^	**
	Sign.	ns	ns	ns	*	ns	

Different uppercase letters indicate significant differences among storage times within the same formulation and temperature according to Tukey’s HSD test (*p* ≤ 0.05). Significance between storage temperatures at the same sampling time is reported as *, *p* ≤ 0.05; **, *p* ≤ 0.01; ns, not significant.

**Table 4:** Evolution of bergamot-derived phenolic compounds quantified in MBE and detected in enriched olive pâté during storage.

IPC (mg g^−1^ DM)	Storage Period	OP_2.5_	OP_5_
Neoeriocitrin	t0	0.13 ± 0.03	0.18 ± 0.03
	t100 (20 °C)	0.17 ± 0.01	0.16 ± 0.02
	t100 (30 °C)	0.15 ± 0.01	0.14 ± 0.02
	Sign	ns	ns
Naringin	t0	0.18 ± 0.01 ^a^	0.21 ± 0.01 ^a^
	t100 (20 °C)	0.13 ± 0.01 ^b^	0.18 ± 0.01 ^ab^
	t100 (30 °C)	0.14 ± 0.01 ^b^	0.16 ± 0.00 ^b^
	Sign	*	*
Neohesperidin	t0	0.11 ± 0.00 ^a^	0.20 ± 0.00 ^a^
	t100 (20 °C)	0.08 ± 0.00 ^b^	0.14 ± 0.00 ^b^
	t100 (30 °C)	0.09 ± 0.01 ^ab^	0.14 ± 0.01 ^b^
	Sign.	*	**

Small letters indicate significant differences between rows, as assessed by Tukey’s post hoc test. Abbreviations: **, significance at *p* < 0.01; *, significance at *p* < 0.05.

**Table 5:** Microbiological stability of olive pâté formulations during storage at 20 and 30 °C.

Parameter	Time (Days)	20 °C				30 °C			
		CTR	OP_2.5_	OP_5_	Sign.	CTR	OP_2.5_	OP_5_	Sign.
CBT (Log_10_ CFU g^−1^)	0	0.00 ± 0.00	0.00 ± 0.00	0.00 ± 0.00	ns	0.00 ± 0.00	0.00 ± 0.00	0.00 ± 0.00	ns
	100	0.30 ± 0.00 ^a^	0.00 ± 0.00 ^b^	0.00 ± 0.00 ^b^	**	0.74 ± 0.10 ^a^	0.30 ± 0.00 ^b^	0.00 ± 0.00 ^c^	**
	Sign.	**	ns	ns		**	ns	ns	
Yeast (Log_10_ CFU g^−1^)	0	0.00 ± 0.00	0.00 ± 0.00	0.00 ± 0.00	ns	0.00 ± 0.00	0.00 ± 0.00	0.00 ± 0.00	ns
	100	1.50 ± 0.12 ^a^	1.32 ± 0.02 ^b^	1.32 ± 0.02 ^b^	**	1.84 ± 0.10 ^a^	1.52 ± 0.02 ^b^	1.32 ± 0.02 ^c^	**
	Sign.	**	**	**		**	**	**	

Different lowercase letters indicate significant differences among formulations at the same storage time and temperature, according to Tukey’s post hoc test. Abbreviations: **, significance at *p* < 0.01; ns, significance at *p* > 0.05.

## Data Availability

The original contributions presented in the study are included in the article. Further inquiries can be directed to the corresponding authors.
